# Effect of upright posture on tonsillar level in adolescent idiopathic scoliosis

**DOI:** 10.1186/1748-7161-10-S1-O51

**Published:** 2015-01-19

**Authors:** Winnie CW Chu, Ryan KL Lee, James F Griffith, Joyce HY Leung, Tsz-Ping Lam, Bobby KW Ng, Jack CY Cheng

**Affiliations:** 1 Department of Imaging and Interventional Radiology, The Chinese University of Hong Kong, Hong Kong; 2 Department of Orthopedics and Traumatology, The Chinese University of Hong Kong, Hong Kong

## Introduction

In the supine position, the cerebellar tonsils in adolescent idiopathic scoliosis (AIS) patients are more low-lying than normal subjects. There is also a positive association between tonsillar descent in AIS patients and impairment of somatosensory cortical evoked potentials (SSEP). In this study, we investigated the effect of standing on tonsillar position in AIS patients and normal controls.

## Aim

To investigate the effect of upright position on tonsillar level in patients with adolescent idiopathic scoliosis (AIS).

## Methods

25 patients with clinically diagnosed AIS (all females, mean age 14.9+/-2.3) and 18 normal age-matched controls (all females, mean age 15.3+/-3.4) were examined by 0.25T MRI (G-Sscan, Esaote, Italy) in both the supine and upright positions. The level of the inferior cerebellar tonsil tip relative to a reference plane connecting the basion to the opisthion (BO line) was measured in millimeters. A position above and below the BO line was assigned a positive value (i.e. no tonsillar descent) and a negative value (i.e. tonsillar descent) respectively.

## Results

In AIS patients, the cerebellar tonsil position was significantly lower in the upright than the supine position (-0.7+/-1.5 vs +1.2+/-1.7, p<0.00001) while in normal subjects, there was no change in cerebellar tonsil position between upright and supine positions (mean +2.1+/-1.7 vs +2.2+/-1.8, p = 0.93). AIS patients also had a greater degree of tonsillar excursion between upright and supine positions compared to normal subjects (mean -1.9 +/-1.6 vs -0.1+/-1.7, p<0.00001). More AIS patients tended to have tonsillar descent in the upright position rather than the supine position though this did not reach statistical significance (28% in supine position vs 48% in standing position, p=0.15). There is slightly better correlation between tonsillar level and Cobb angle in upright position (r = 0.45, p = 0.01) than those in supine position (r= 0.31, p=0.03) in AIS patients.

## Conclusion

AIS patients have more tonsillar descent in the upright position and a greater degree of tonsillar excursion between supine and upright positions compared to matched control subjects. Apart from supporting the hypothesis of relative cord tethering in AIS, the results also enhance the likelihood of more significant chronic dynamic compression of the brainstem and upper cervical cord in AIS. Whether this will lead to chronic insult with subclinical neurophysiological disturbance, affecting both dynamic postural balance and SSEP, and contributing to the etiopathogenesis of AIS, warrants further study.

